# Randomised, double-blind comparison of a fixed co-formulation of intra-articular polynucleotides and hyaluronic acid versus hyaluronic acid alone in the treatment of knee osteoarthritis: two-year follow-up

**DOI:** 10.1186/s12891-021-04648-0

**Published:** 2021-09-12

**Authors:** Cesare Stagni, Martina Rocchi, Alessandro Mazzotta, Nicolandrea Del Piccolo, Nicola Rani, Marco Govoni, Leonardo Vivarelli, Francesca Veronesi, Milena Fini, Dante Dallari

**Affiliations:** 1 Reconstructive Orthopaedic Surgery and Innovative Techniques — Musculoskeletal Tissue Bank, IRCCS Istituto Ortopedico Rizzoli, Via G.C. Pupilli 1, 40136 Bologna, Italy; 2 Surgical Sciences and Technologies Complex Structure, IRCCS Istituto Ortopedico Rizzoli, Via di Barbiano 1/10, 40136 Bologna, Italy

**Keywords:** Knee osteoarthritis, Knee function, Hyaluronic acid, KSS, Knee pain, PN-HPT™, Polynucleotides, WOMAC

## Abstract

**Background:**

A first-year interim analysis of this two-year study suggested that intra-articular injections of highly purified, natural-origin polynucleotides and hyaluronic acid (HA) as a fixed combination (PNHA) might improve knee function and joint pain more effectively than HA alone in patients with knee osteoarthritis (OA). The purpose of the second-year analysis herein described was to verify whether the first-year interim outcomes persist over the whole two-year period.

**Methods:**

Randomised, double-blind, HA-controlled clinical trial in 100 knee OA patients (98 randomised, 79 completing the study) in a high-specialisation tertiary care setting. The hypothesised difference of efficacy between PNHA and HA for the original sample size estimate is 20%. Treatment cycle: three intra-articular knee injections of either PNHA or HA, at baseline and weekly for two weeks. Evaluations: Western Ontario and McMaster Universities (WOMAC) score and Knee Society Score (KSS) as, respectively, primary and secondary endpoints, evaluated at baseline and after 2, 6, 12, and 24 months; synovial fluid levels of mediators (at baseline and the end of the treatment cycle). Adverse effects investigated at each control visit. Statistical analysis: Kruskal-Wallis test for independent samples (nonparametric one-way analysis of variance) after correction of means for age, Body Mass Index and Kellgren-Lawrence grade. If significant, pairwise post-hoc Sidak multiple comparisons.

**Results:**

KSS total score and KSS pain item: significant improvement in both groups, with significantly more pain improvement in patients treated with PNHA (2-point reduction) than HA (1-point reduction). Both groups experienced significant long-term reductions in WOMAC total scores: significantly stronger in PNHA-treated patients after 24 months with a steady difference of 16% favouring PNHA in WOMAC pain subscore. No clinically significant adverse events in either group.

**Conclusions:**

The outcomes of the 2-year study confirmed that a short cycle of intra-articular treatment (3 weekly double-blind injections) with polynucleotides (long-acting viscosupplementation properties, chondrocyte activation, pain-relieving properties) in fixed combination with high molecular weight hyaluronic acid is more effective in improving knee function and pain in knee OA patients than HA alone. PNHA may be elective for viscosupplementation in knee OA patients with fastidious and resistant pain and worsening disease.

**Trial registration:**

NCT02417610.

Registration, 15/04/2015.

ClinicalTrials.gov database link:

## Background

The debate about the actual value of hyaluronic acid (HA) as infiltrative therapy of knee osteoarthritis (OA) is far from over in evidence-based guidelines and consensus reports [1–3]. Highly purified polynucleotides from trout gonads, also known with the acronym PN-HPT™ (Polynucleotides Highly Purified Technology), provide persistent viscosupplementation, provide nitrogen bases and nucleoside and nucleotide precursors to chondrocytes and mesenchymal cells, and reduce pain more effectively and more rapidly than HA [4–6]. In-vitro and in-vivo synergy between PN-HPT™ and HA on chondrocyte trophism and pain control has also been convincingly established—a solid rationale for administering the two viscosupplementation agents together [7].

The study aimed to verify over 2 years whether the association of PN-HPT™ and HA injections would reduce pain in patients affected by knee OA more than HA alone, and whether it is more effective in improving knee function and pain, in joints affected by OA, compared with HA alone, as suggested by the authors in their first-year interim report [8]. The final two-year outcomes of the study also aimed to verify whether the clinical synergy between PN-HPT™ and HA, which the first-year interim analysis suggested, is persistent over a much longer time or just a transient medium-term effect.

## Methods

### Study design, sample size estimate, inclusion and exclusion criteria

Randomised, double-blind, single-centre, HA-controlled clinical trial in a high-specialisation tertiary care setting. A hundred knee OA patients, aged between 51 and 74 years, were initially screened between mid-September 2014 and mid-July 2015, and 98 randomised.

The inclusion and exclusion criteria and the demographics of the screened knee OA patients are summarised in Table 1 and Table 2, respectively. Two screened patients were excluded after failure to meet the inclusion criteria at a secondary control. The authors carried out the study at IRCCS Istituto Ortopedico Rizzoli, Bologna, Italy, following the most recent clinical practice guidelines and ethical regulations (for details, see the report that discussed the interim outcomes after the first year of treatment) [8]. The final, two-year outcomes are herein illustrated. The intake of NSAIDs and other drugs was free during the two-year study period; investigators only recorded the NSAIDs consumption since the last visit.

**Table 1:** The inclusion and exclusion criteria adopted for selecting the 98 enrolled patients [8]

Inclusion criteria	
Individuals of both genders aged between 50 and 75 with knee osteoarthritis according to the American College of Rheumatology (ACR) criteria	
At least five-year schooling to be able to give written informed consent properly	
Kellgren-Lawrence (KL) knee OA severity grade: between 1 and 4 [ 9]	
Current pain that has persisted for at least two months	
Body Mass Index (BMI) at screening ≤40 kg/m [2]	
Exclusion criteria	
Abuse of alcohol or drugs	
Pregnancy or breastfeeding	
Patients who underwent repeated infiltrative therapies suspended from less than three months	
Ongoing treatment with systemic anticoagulants or steroids, or therapy suspended for less than one month	
Hypersensitivity to the study products, previous bone fractures, severe knee trauma, joint deformities, rheumatoid arthritis, inflammatory diseases of joints, other current severe diseases, previous surgical procedures (e.g., meniscectomy, scope debridement)	
Haematological diseases or local skin lesions in the site of treatment inoculation

**Table 2:** Demographics of the initially screened knee osteoarthritis patients [8, 9]

	All patients (*n* = 100)	Study Group (PNHA, *n* = 49)	Control Group (HA, n = 49)
Age, yrs	50–75 (63.8 ± 5.8)	63.4 ± 6.5	64.2 ± 5.1
Kellgren-Lawrence grade	2 ± 0.7	1.9 ± 0.6	2.1 ± 0.7
Sex, male/female, n	46/54	24/26	22/28
Body Mass Index, kg/m^2^	28,1 ± 3,5	28,1 ± 3,4	28,1 ± 3,7
Weight, kg	80.0 ± 11.6	80.2 ± 10.2	79.8 ± 13
Height, cm	168,5 ± 9.2	168,9 ± 9.5	168,1 ± 9.0

The assumptions initially leading to the sample size calculation and the technicalities adopted for creating the randomisation list and preserving the double-blindness of all those involved, patients, investigators, data collectors and outcome assessors, were exhaustively described in the first-year interim report [8].

The main points about the sample size estimate are herein summarised. With the per cent WOMAC change at 12 months considered as the primary endpoint, the following formula gave an estimate of the needed sample size: [8]

$$ \Delta \mathrm{WOMAC}\ \left(\mathrm{per}\ \mathrm{cent}\ \mathrm{difference}\ \mathrm{vs}\ \mathrm{baseline}\right)=\frac{12-\mathrm{month}\ \mathrm{WOMAC}-\mathrm{baseline}\ \mathrm{WOMAC}}{100-\mathrm{baseline}\ \mathrm{WOMAC}} $$

Based on previous HA literature and exploratory unpublished PNHA minor studies, the basic assumption leading to the original sample size estimate was that standard deviations were 26.9% for PNHA-treated patients and 39.1% for HA-treated patients. Further assumptions were that standard deviations would be similar for the two populations to be enrolled. At the same time, the test would aim to detect a clinical efficacy difference between the two intrarticular treatments of at least 20% under the null hypothesis that the two treatments had similar WOMAC per cent variations. With the assumption of a false-positive (alpha) error of 0.05 and power to avoid false negatives of at least 0.80, a minimum clinically meaningful difference of 20% and a drop-out rate of 10%, the minimum estimated number of patients was 50 per group (100 overall) [8]. Software used for statistical analyses: IBM SPSS Statistics 21.

The coded packages of PNHA and HA syringes were identical, with syringes masked by identical sleeves. The randomisation list reported the numerical code on syringe packages; the investigators received the randomisation codes for each patient sealed in an envelope [8].

Ninety out of initially randomised patients completed the study at [T5] (interim evaluation after the first year of treatment), 46 in the PNPHA study group and 44 in the HA control group; all of them then progressed to [T6] (end of study). Seventy-nine patients completed the 2-year study. All the patients who had dropped out at the end of the first year did it for personal reasons [8].

### Treatments

The regulatory classification of the patented, proprietary fixed PNHA combination investigated in the 2-year study was as a Class-III CE-marked (0373) medical device: pre-filled, single-use, neutral glass 2-mL syringes dosed at 10 mg/mL of natural-origin PN-HPT™ and 10 mg/mL of a biotechnological sodium HA (molecular weight > 1500 kDa) for an overall syringe content of 40 mg in 2 mL of active principles. The European Union’s regulatory authorities and several extra-European countries registered the proprietary fixed PNHA combination (brands according to countries, POLIART^®^ and CONDROTIDE Plus^®^, Mastelli Srl, San Remo, Italy; indication “intra-articular treatment of degenerative chondral disorders”). The control HA product (IALART^®^, Mastelli Srl, San Remo, Italy) is also a Class III CE 0373 commercially available medical device of HA (1200–1500 kDa), industrially obtained from bacterial fermentation and dosed at 40 mg in 2 mL. The formulation of both study products was as absorbable, viscoelastic sterile gels.

**Fig. 1:**
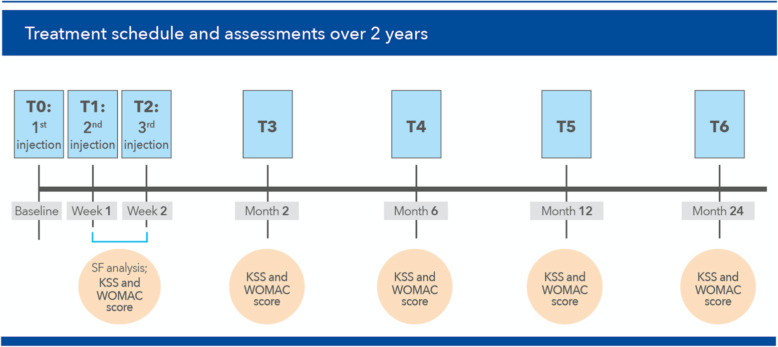
[T0] to [T2]: timing of the three PNHA and HA intra-articular injections and assessments planned over the first two study weeks (synovial fluid analysis and first KSS and WOMAC evaluation); [T3] to [T6]: timing of the KSS and WOMAC evaluations planned over the residual 2-year study period

Highly skilled specialists performed three weekly intra-articular double-blind infiltrations with 18 to 22 G needles at baseline [T0] and over the following two weeks [T1] and [T2], under aseptic conditions and following standard intra-articular techniques (injected amount at each session, 2 mL). Samples of the synovial fluid (nearly 6 mL of the removed excess synovial fluid) were collected and sent to the laboratory before the first infiltration [T0] and at the end of the treatment cycle [T2] (Fig. 1).

### Follow-up assessments

The knee joint function and pain were assessed, with the help of the Knee Society Score (KSS) [10] and the self-administered Western Ontario and McMaster Universities (WOMAC) score [11], at baseline [T0] and after 2 [T3], 6 [T4], 12 [T5] and 24 months [T6] during the 2-year follow-up. A radiographic examination complemented the final clinical evaluation at [T6]. The WOMAC pain subscore was the primary endpoint; KSS, the overall WOMAC score and NSAID consumption were secondary endpoints. Assays of the viscosity of the synovial fluid and the synovial fluid levels of some markers associated with cartilage damage such as matrix metalloproteinase-1 (MMP1), MMP13, tissue inhibitor of MMP1 (TIMP1) were also planned in 40 patients. Assays timing: baseline [T0] and the end of the 2-week treatment cycle [T2] using standard immunoenzymatic assays (complete technical details of commercial assays and procedures are available in Ref. [8]. As far as possible, all WOMAC and KSS scoring, and indeed all clinical evaluations and assays on synovial fluid, were performed by the same investigator with only a very few exceptions. At each follow-up visit, investigators recorded all local and systemic side effects in the electronic clinical report form, and the casual relationship immediately assessed and reported for further evaluation.

### Statistical analysis

Descriptive data were tabulated as means ± standard deviations (SD) and graphically as boxplots. The general linear model for repeated measures or Kruskal-Wallis test for independent samples (nonparametric one-way ANOVA test) was applied, after correction of means for age, BMI and KL grade [12], to assess how the treatments influenced the follow-up curves. Using the nonparametric one-way ANOVA test was justified because data (WOMAC, KSS, KSS subscore for pain) were not continuous, although variance was homogeneous (Levene’s test). After detecting significant effects of treatments, pairwise post-hoc Sidak multiple comparisons identified the exact time points of divergence of the curves during the [T3] to [T6] follow-up period.

Regarding the synovial fluid analyses (already reported in the first-year report), the t-test for paired samples (one-sample t-test) was used to compare between experimental times within groups and the unpaired t-test (two-sample t-test) for comparisons between groups. The Pearson test for linear relationships between two continuous variables was used to investigate the correlations between the synovial markers, both among them and between them, and the KSS or WOMAC scores at [T0] and at the end of treatment—[T2] for SF and [T3] for KSS and WOMAC scores. Further statistical details are available in Ref. 8.

### Ethical considerations

The Institutional Review Board of Istituto Ortopedico Rizzoli reviewed all study materials for ethical problems, and the principles of the Declaration of Helsinki were always respected. The study was registered in the ClinicalTrials.gov database of privately and publicly funded clinical studies conducted worldwide (ClinicalTrials.gov Identifier: NCT02417610).

## Results

**Fig. 2:**
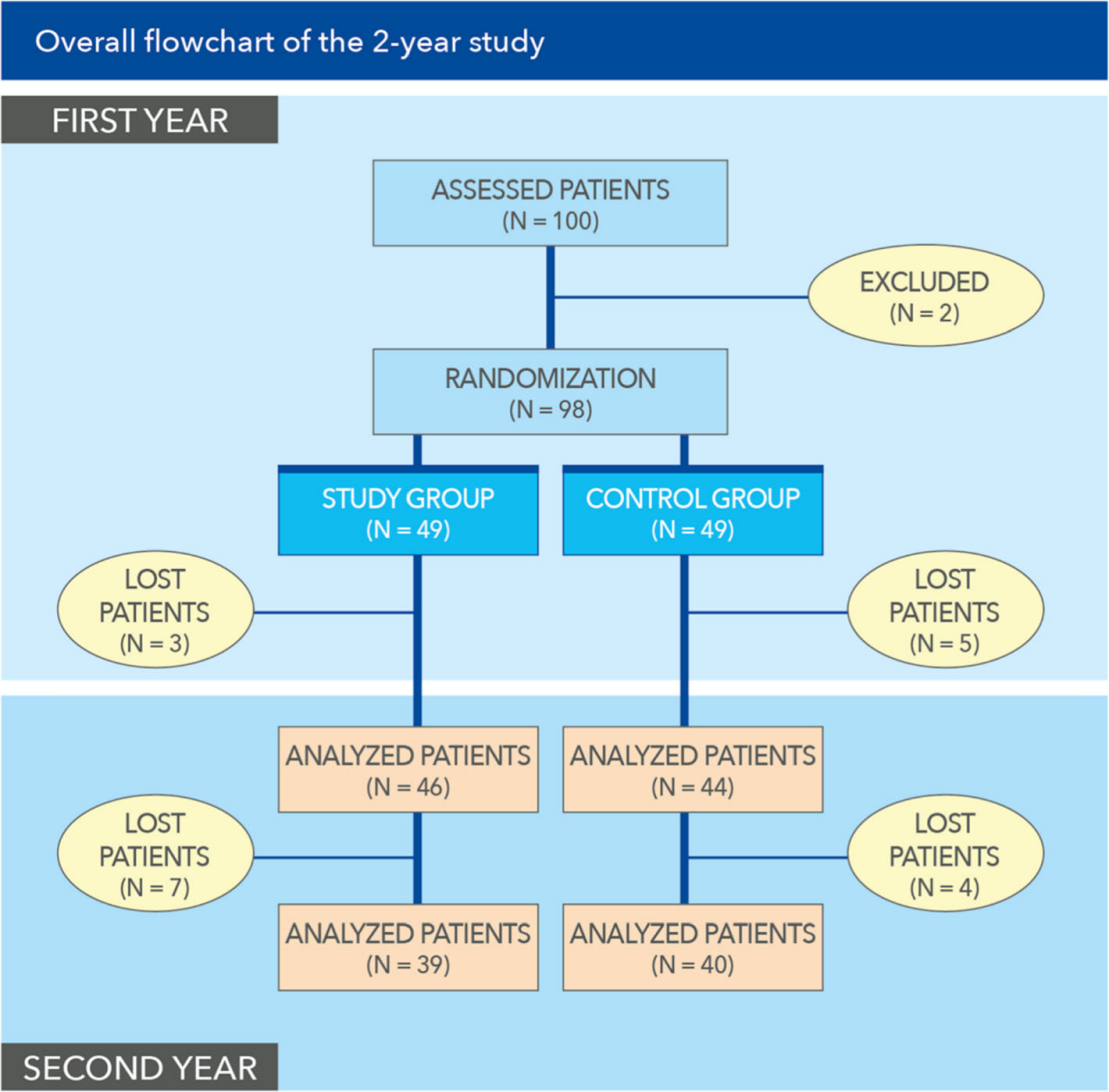
*Upper lighter blue area:* first-year part of the study leading to the interim analysis at the end of the first study year—i.e., outcomes up to 12 months [T5] discussed in Ref. 8. *Lower darker blue area:* second-year follow-up

Figure 2 illustrates the overall flowchart of the 2-year study. At [T5], the patients of the two groups who progressed towards T6 and the end of the study were still homogeneous for age (*p* = 0.54), Kellgren–Lawrence grade (*p* = 0.13), gender (*p* = 0.84), BMI (*p* = 1), weight (*p* = 0.86), and height (*p* = 0.67).

**Fig. 3:**
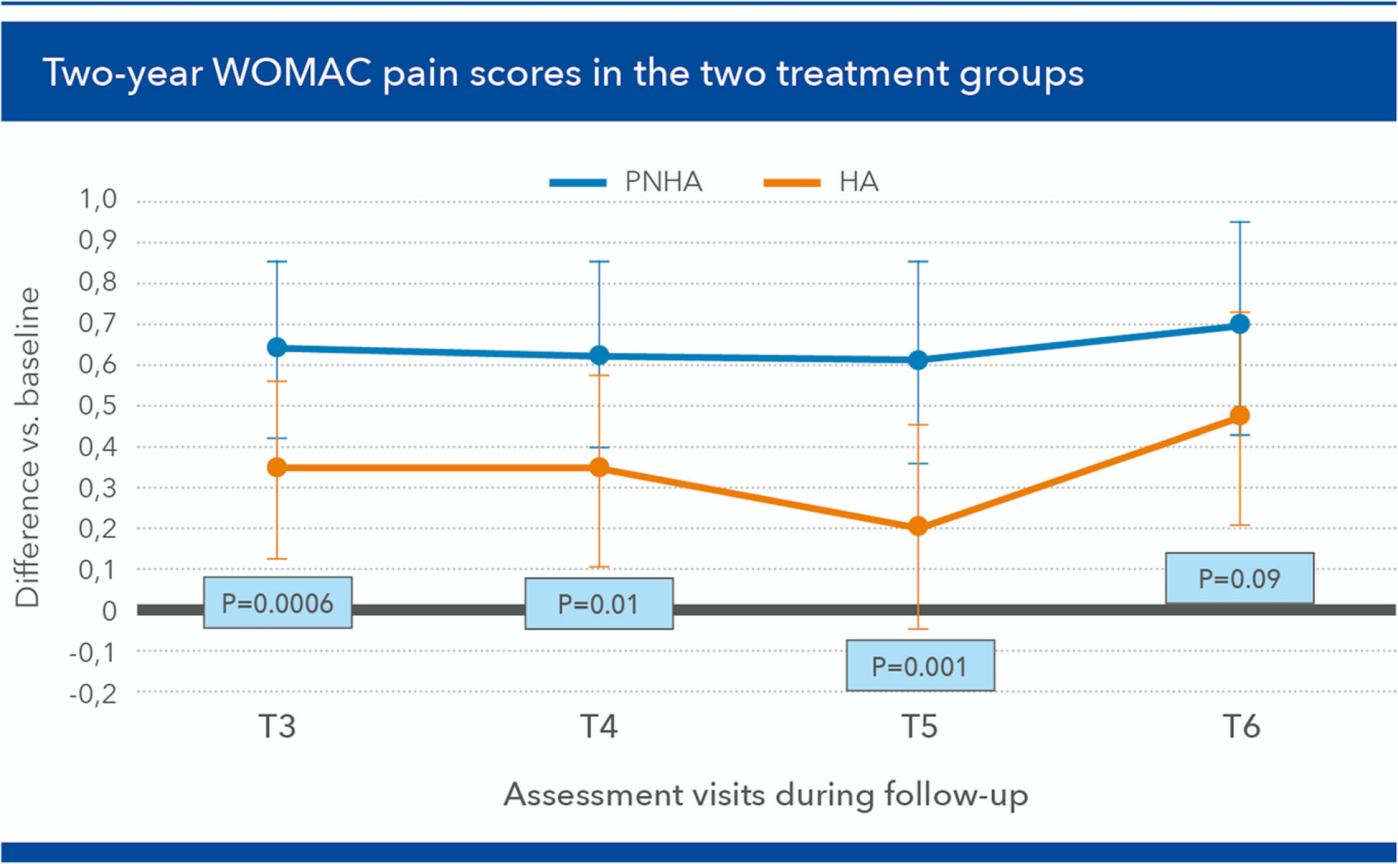
Differences in Western Ontario and McMaster Universities (WOMAC) pain scores (primary endpoint; mean ± SD) vs baseline during the [T3] (2 months) to [T6] (24 months) follow-up period (positive values: improvement vs baseline)

As reported in the interim report, the first year of follow-up saw no infiltration-related complications [8]. Seventy-nine patients completed the study (39 in the PNHA group, 40 in the control HA group), with seven more patients lost in the PNHA group and four in the HA group, once again due to personal reasons. As regards the primary endpoint, WOMAC pain score, the pain curves were significantly different at one-way ANOVA (*p* = 0.029; partial eta squared = 0.07); divarication of pain curves was both precocious, (already demonstrable at [T3], *p* = 0.0006 at Sidak test, then steady for 2 years—[T4] *p* = 0.01, [T5] *p* = 0.001, [T6] *p* = 0.09 (Fig. 3).

**Fig. 4:**
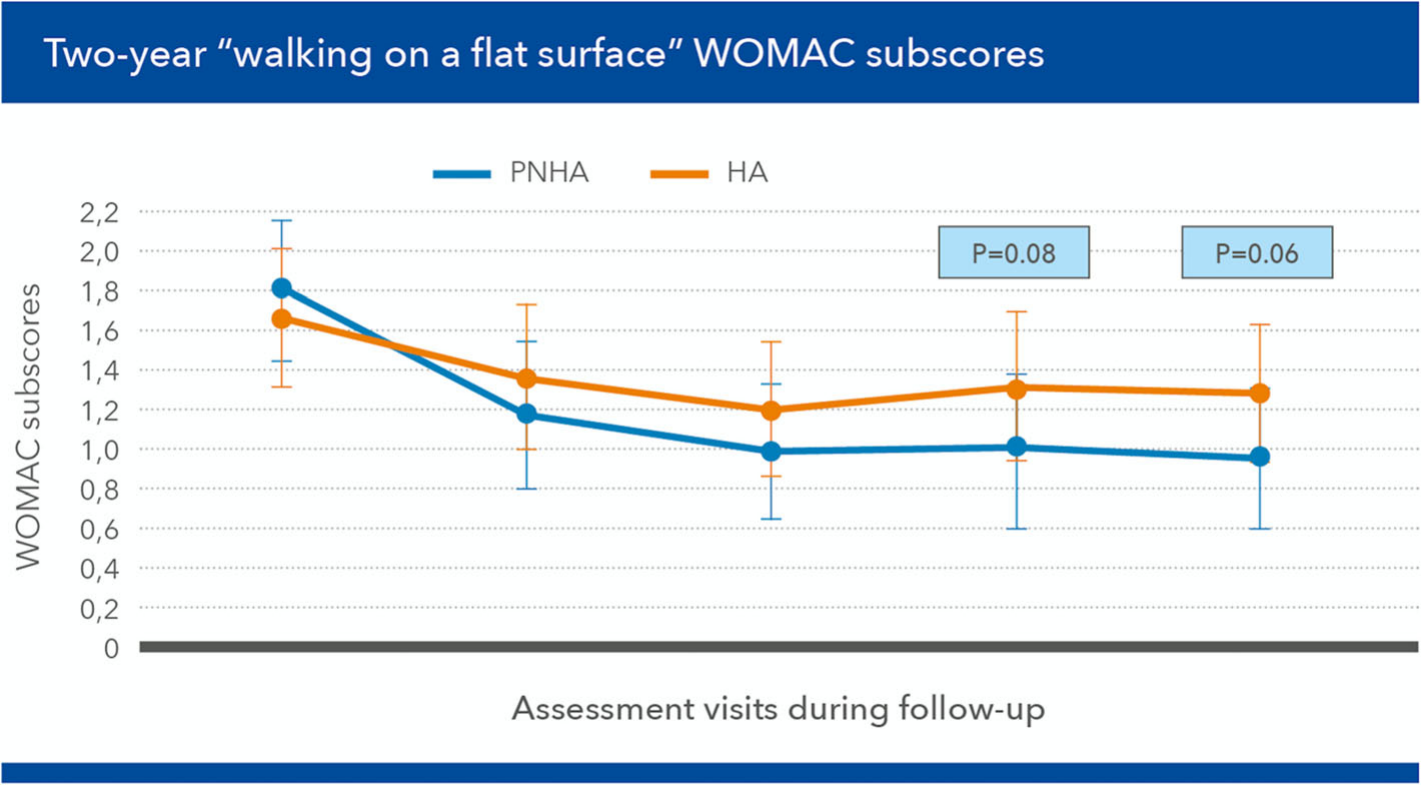
Mean “walking on a flat surface” Western Ontario and McMaster Universities (WOMAC) subscores (mean ± SD) during the [T3] (2 months) to [T6] (24 months) follow-up period

**Fig. 5:**
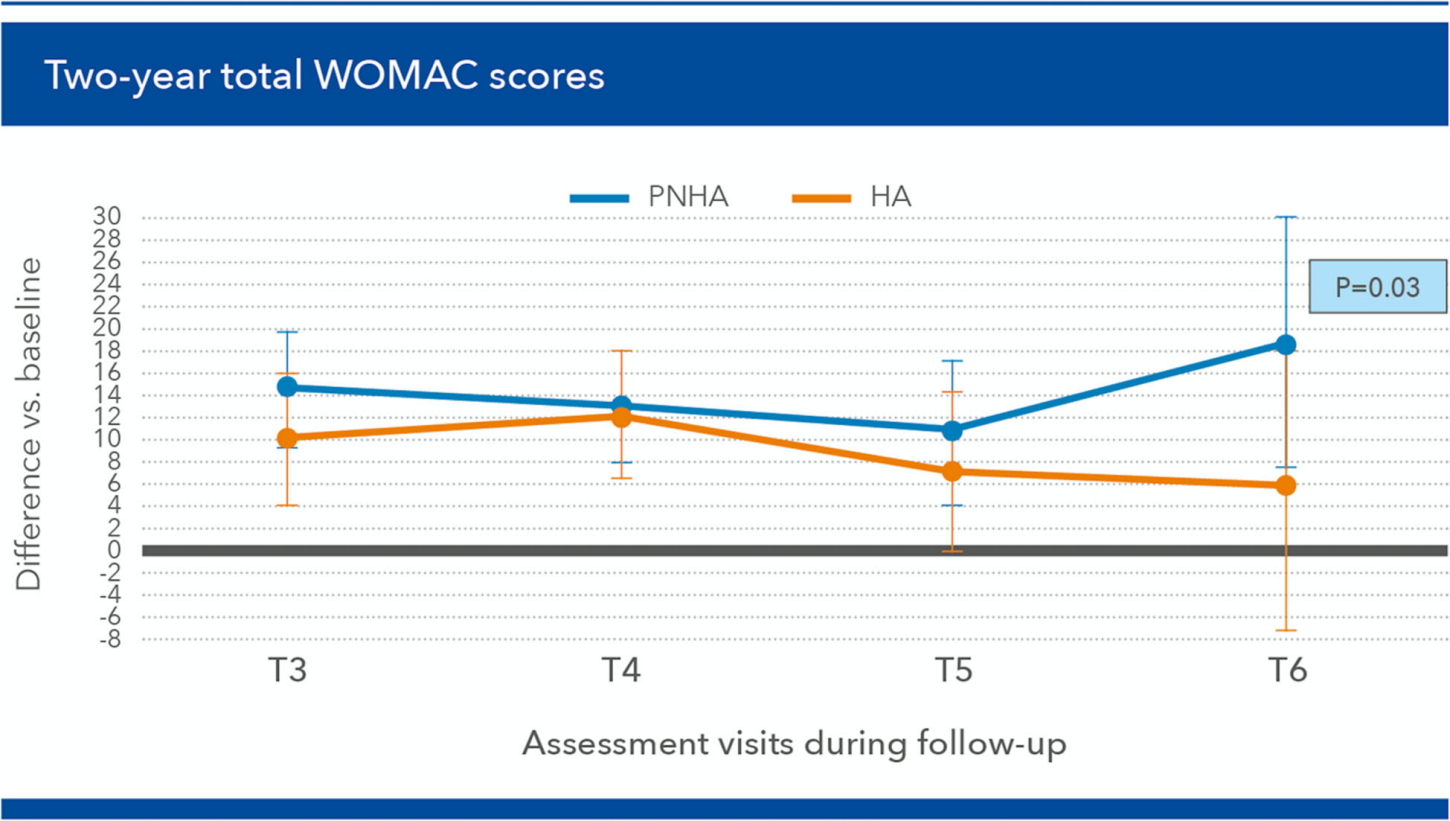
Differences in total Western Ontario and McMaster Universities (WOMAC) scores (mean ± SD) vs baseline during the [T3] (2 months) to [T6] (24 months) follow-up period (positive values: improvement vs baseline)

The mean difference in favour of the PNHA group vs the HA control group was about 16% over the 2-year follow-up period. The pain improvement showed significant differences at [T4] (*p* = 0.029) and [T5] (*p* = 0.046), and an almost significant difference at [T6] (*p* = 0.059). The other WOMAC items did not show differences between the two groups, with the partial exception of “walking on a flat surface”, which was always tendentially easier for patients in the PNHA group and significantly so at [T5] and [T6] (Fig. 4). As a result, the mean total WOMAC scores showed a tendency to improve steadily more in the PNHA group than HA controls, over the whole follow-up period (Fig. 5), although the difference was statistically significant only at [T6] after corrections for age and other parameters.

**Fig. 6:**
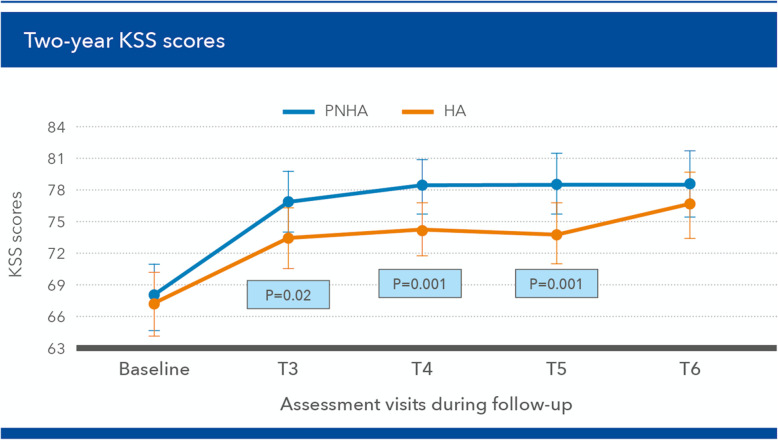
Knee Society Score (KSS) scores (mean ± SD) during the [T3] (2 months) to [T6] (24 months) follow-up period

The KSS total scores over the first year were always significantly higher in the PNHA study group compared with the HA control group at all follow-up assessments (*p* = 0.02 at [T3] and *p* = 0.001 at both [T4] and [T5]). The 2-year study confirmed the tendency towards a long-term pain benefit for PNHA-treated patients at the last [T6] assessment (Fig. 6).

**Fig. 7:**
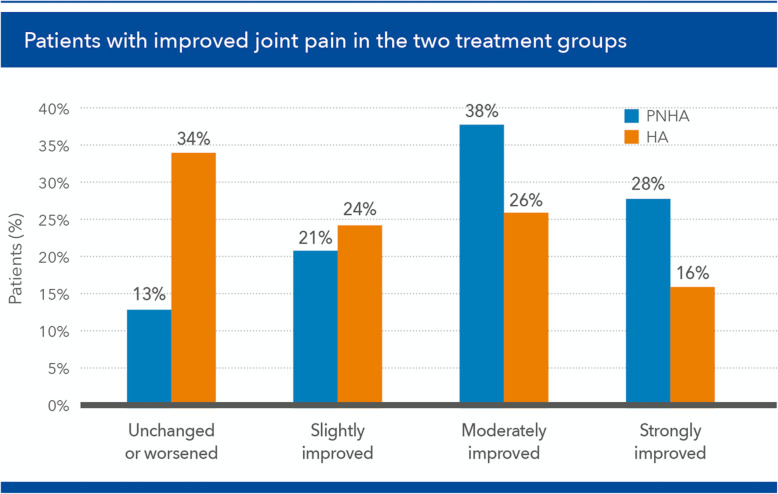
Percent of patients in the fixed combination (PNHA) and hyaluronic acid (HA) treatment groups reporting improvement in Knee Society Score (KSS) pain scores during the [T3] (2 months) to [T6] (24 months) follow-up period

The overall outcomes were similar for the KSS “pain” item subscore (*p* < 0.05 at [T3] and [T5]; [T6] *p* = 0.059 marginally not significant), with 87% of patients of the PNHA treatment group (34 out of 39) and 66% of the HA group reporting an improvement of joint pain (Fig. 7).

**Fig. 8:**
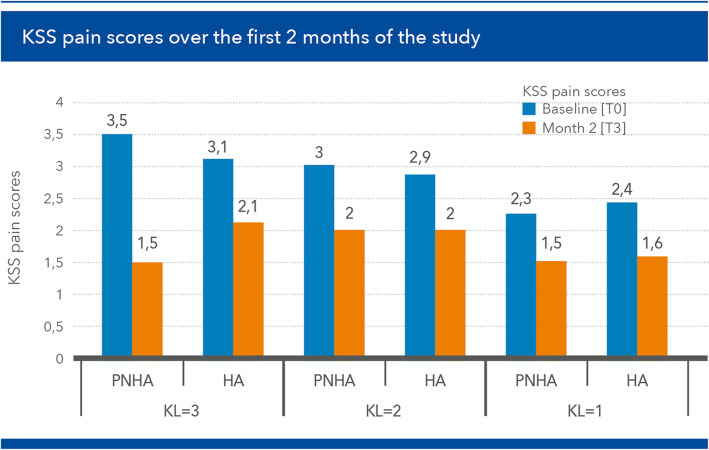
Mean Knee Society Score (KSS) pain scores at baseline and [T3] (2 months) in patients of the fixed combination (PNHA) and hyaluronic acid (HA) treatment groups according to baseline severity (Kellgren–Lawrence grade) of knee joint disease

The degree of improvement in mean KSS pain scores was different in patients of the PNAH treatment group and patients of the HA group as a function of joint damage severity, with a more substantial decrease of pain scores in patients with more severe disease (Fig. 8).

**Fig. 9:**
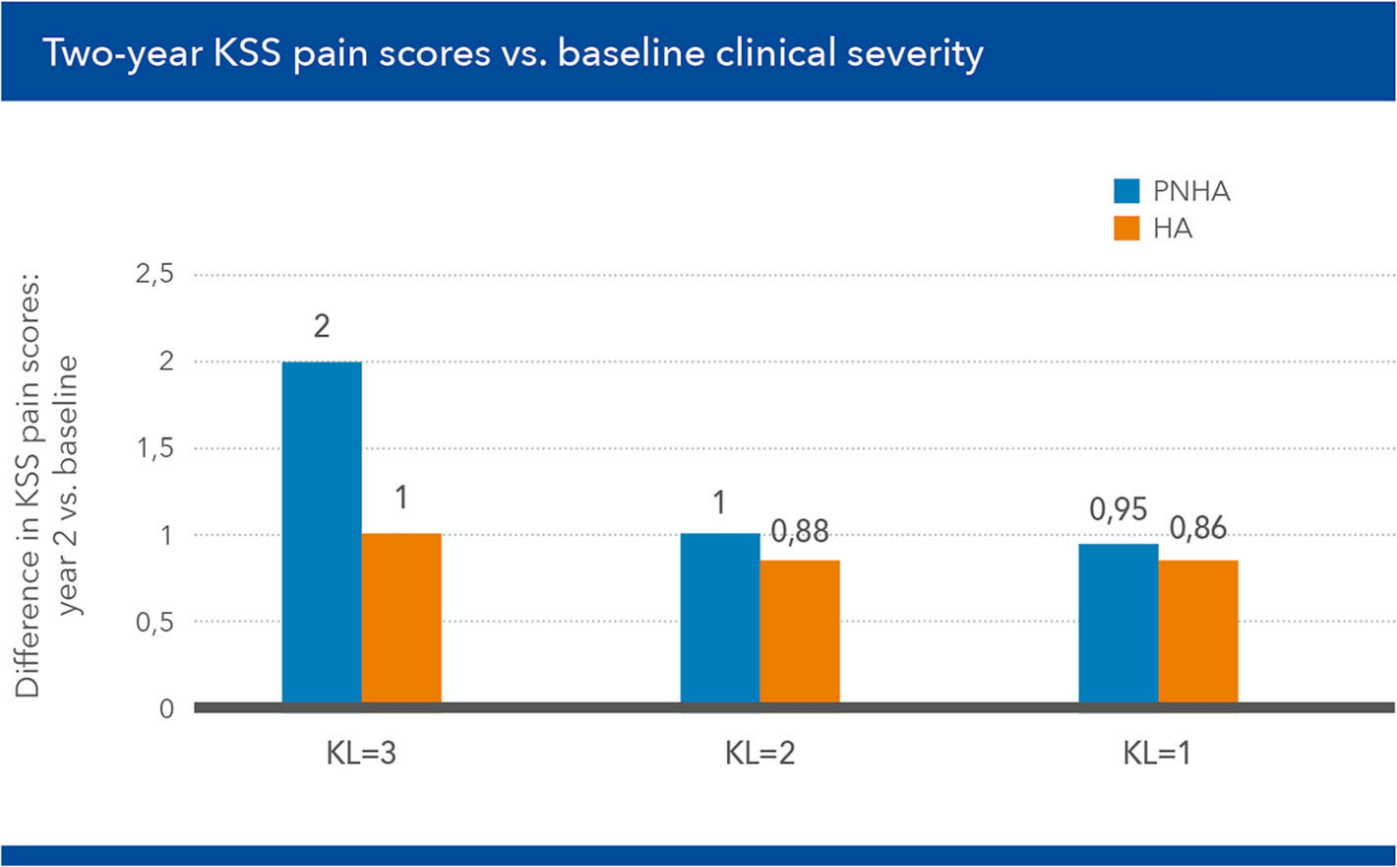
Improvement in mean Knee Society Score (KSS) pain scores, baseline vs [T6] (24 months) in patients of the fixed combination (PNHA) and hyaluronic acid (HA) treatment groups according to baseline severity (Kellgren–Lawrence grade) of knee joint disease

Mean KSS pain scores improved by 2 points both early after the end of the treatment cycle [T3] and at the end of the 2-year follow-up [T6] in PNHA-treated patients with more severe knee joint disease; conversely, KSS pain scores improved by 1 point in the HA-treated patients with the same degree of disease severity (Fig. 9). Mean improvements were similar in patients with less severe disease; NSAIDs consumption was similar in the two treatment groups (11 patients in both groups).

The synovial fluid samples of all patients were transparent or translucent, showed a well-defined clot, and were of a regular yellow or, more frequently, light yellow colour. The synovial fluid clarity and density (mucin clot test) were also in the expected range in all patients. The total white cell count was always within the non-inflammatory range of less than 2000 cell/mm^3^

As already discussed in the first-year report, looking for some short-term correlation between symptoms and the synovial fluid levels of soluble factors related to joint gave no significant results [8]. MMP1 and MMP13, showed some tendency to fall, on average after two months of PNHA treatment compared with baseline, after 2 months of PNHA treatment (− 49 ± 27% for MMP1; − 31,2 ± 21,3% for MMP13). Conversely, MMP1 and MMP13 levels tended to increase (+ 29,5 ± 16,7% and + 6 ± 3,7%, respectively), but without statistical correlations with symptom evolution.

Neither infiltrative treatment was associated with short-term complications or long-term side effects of any clinical significance.

## Discussion

The final two-year outcomes of this randomised, double-blind study confirm the preliminary outcomes of the previous 1-year interim report—the intra-articular co-administration of a fixed combination of PN-HPT™ and HA is associated with significant benefits for the knee joint pain, the primary study endpoint, and functional disabilities compared with HA alone [8]. The final two-year outcomes of the study also support the rationale that inspired the development of the fixed PNHA combination—synergy between PN-HPT™ and HA is likely in OA based on the complementary properties of the two viscoelastic agents [8].

Highly purified, natural-origin PN-HPT™ — linear chains of polynucleotides from trout gonads — release nucleosides, nucleotides, and nitrogen bases by enzymatic cleavage in the synovial space and have shown long-term moisturising and viscoelastic properties in clinical studies in knee OA [4–6]. PN-HPT™ may combine their primary viscolelastic and mechanical properties with some indirect protecting and possibly trophic activity on chondrocytes, fibroblasts and cartilage by providing physiological precursors [7, 12]. Such PN-HPT™ indirect activity appears stronger than HA, supporting the “PN-HPT™ plus HA” synergy concept, mainly based on viscoelastic and mechanic properties, that inspired the 2-year study herein discussed and the first-year interim report [8]. PN-HPT™ also seem to be associated with more substantial pain-reduction properties than HA in patients with knee OA [4].

The more rapid reduction of WOMAC pain scores, the primary endpoint of the study, in PNHA-treated patients compared with the HA group is likely to mirror the synergic short-term viscoelastic contribution of PN-HPT™ to the investigated fixed formulation. Such viscoelastic synergy also likely explains the steady long-term reduction of knee pain, substantial at [T4] and [T5] (months 6 and 12) compared with HA-treated patients, but extending over the whole two-year study period. Without that synergy in the HA treatment group, pain significantly decreased only at the second and fourth months of follow-up ([T3] and [T4]), but not after 12 ([T5]) and 24 months ([T6]). Of course, using a low-molecular-weight HA in controls, which may have low elastoviscosity and require frequent infiltrations, might have acted as a confounding factor tht deserves remark [13]. The benefits for the WOMAC item “Walking on a flat surface” developed somewhat more slowly in PNHA-treated patients, with still no differences between PNHA and HA at [T3] and [T4], but statistically significant ones at both [T5] and [T6].

The PN-HPT™ viscoelastic contribution might have helped to improve knee OA symptoms more effectively and possibly earlier than HA in patients with high-grade chondropathy, as shown in previous observations [5, 6]. More specifically, the PNHA treatment group experienced more substantial two-year reductions of both KSS and, even more, WOMAC mean pain subscores than the HA treatment group. Pain benefits, already manifest in patients with the least severe disease (KL grade 1), increased progressively with disease severity, from KL grade 1 up to KL grade 3. The lack of correlation between symptom severity and the synovial levels of soluble markers related to joint damage prevents casting some light on such reasonable pain control despite advanced joint damage [14, 15].

A retrospective stratification of OA severity supports the former observation about the comparative pain benefits progressively increasing in grade-1, grade-2 and grade-3 OA patients. However, the observation is limited to pain, meaning caution is warranted.

The one-year interim report discussed the rationale for assaying intra-articular factors related to joint damage to cast some light on the still debated association between pain and synovial fluid markers [8, 16–18]. Indeed, the authors only found a feeble inverse correlation between the total KSS score and reduced MMP1 and MMP13 synovial levels in the PNHA treatment group. Such observations confirm the basically viscoelastic and mechanic PN-PHT™ action>, although the high inter-individual variability the low number of synovial fluid samples and the short treatment period may have acted as confounding factors. As stated in the previous interim report, detecting clinically relevant differences in synovial fluid inflammatory markers might have required more follow-up time after the treatment cycle and more synovial fluid samples [8].

As a final consideration, the authors acknowledge some weak points of their study: for instance, a three-edged, parallel-group study — placebo, PN-HPT™, PNHA — would have been more discriminating and informative. The primary study purpose was to identify a role, if any, and possibly a therapeutic niche for PNHA in the current HA-dominated landscape, and this justified the two-group design, according to the authors. The authors feel the study fulfilled this limited goal; other considerations, including pharmacoeconomics, will have to wait for future studies. The low mean clinical severity of enrolled patients (2 ± 0.7 for all patients, 1.9 ± 0.6 for the PNHA group) is possibly another weak point. Incorporating more grade-3 patients would have been likely more discriminating in a study of such ambition.

A third point liable to criticism: why falling back to traditional radiology instead of evaluating cartilage trophism with a rapid magnetic resonance imaging technique like 3 T MRI? The reason was simple: even in an excellence centre, the risk of overburdened MRI resources was steadily substantial over the study years. A fourth methodological weak point is the lack of confidently effective methods to assess and control compliance. Investigators went to great lengths to discuss with all patients in the treatment groups how compliance is crucial. However, the lack of in-depth compliance controls, based on mechanical timed counters, might have introduced bias.

Summarising, as shown by the two-year evolution of the primary endpoint, the WOMAC pain score, the study demonstrated a steady, long-term improvement of OA-related knee pain in PNHA-treated patients. The pain benefit vs HA was significant at all assessment times and greater in patients with a high KL degree of basal OA severity. It most probably correlated with the viscoelastic mechanical synergy between PN-HPT™ and HA due to the lack of any real correlation between symptom evolution and synovial soluble markers of joint damage. Conversely, WOMAC pain control was somewhat unsteady in many patients of the HA treatment group, worsening after six months and one year of follow-up, and, at least tendentially, even after two years. Although some secondary endpoints did not show significant differences, KSS pain control was more rapid, already after two months after the end of the treatment cycle, in PNHA-treated patients.

## Conclusions

The two-year, double-blind study outcomes confirmed natural-origin, highly purified polynucleotides (PN-HPT™) as agents with long-acting viscosupplementation and persistent protective activity in chondrocytes, and a valuable complement to HA for the relief of pain and functional symptoms in knee OA. The suggested PNHA therapeutic range is 2 to 4 mL, but even the lowest dose used in the trial (2 mL) led to favourable results. The indirect PN-HPT™ supporting activity on connective tissues suggested by in-vitro studies, including on chondrocytes and the joint cartilage, might be especially of value as the basis of the likely in-vivo synergy between the two viscoelastic agents.

## Data Availability

The datasets generated and analysed during the current study, not publicly available, are currently archived according to current regulations (with full personal details of all participating subjects) at Istituto Ortopedico Rizzoli , Bologna, Italy. All the datasets are available (after conversion in anonymous form) from the corresponding author on reasonable request.

## Abbreviations

3 T MRI: 3-Tesla Magnetic Resonance Imaging
ACR: American College of Rheumatology
BMI: Body Mass Index
CRP: C-Reactive Protein
kDa: kilodalton
E_2_: Prostaglandin E_2_
ESR: Erythrocyte sedimentation rate
HA: Hyaluronic acid
KL: Kellgren-Lawrence grade
KSS: Knee Society Score
MMP1: Matrix Metalloproteinase-1
MMP13: Matrix Metalloproteinase-13
NSAID: Non-Steroidal Anti-Inflammatory Drug
OA: Osteoarthritis
OARSI: Osteoarthritis Guidelines Development Group
PNs: Polynucleotides
PNHA: PNs and HA fixed combination
PN-HPT™: Polynucleotides “highly purified technology”
TIMP: Tissue inhibitor of MMP1
TKA: Total Knee Arthroplasty
WOMAC: Western Ontario and McMaster universities

## References

[1] McAlindon TE, Bannuru RR, Sullivan MC, Arden NK, Berenbaum F, Bierma-Zeinstra SM (2014). OARSI guidelines for the non-surgical management of knee osteoarthritis. Osteoarthr Cartil.

[2] Henrotin Y, Raman R, Richette P, Bard H, Jerosch J, Conrozier T, Chevalier X, Migliore A (2015). Consensus statement on viscosupplementation with hyaluronic acid for the management of osteoarthritis. Semin Arthritis Rheum.

[3] Bannuru RR, Osani MC, Vaysbrot EE, Arden NK, Bennell K, Bierma-Zeinstra SMA, Kraus VB, Lohmander LS, Abbott JH, Bhandari M, Blanco FJ, Espinosa R, Haugen IK, Lin J, Mandl LA, Moilanen E, Nakamura N, Snyder-Mackler L, Trojian T, Underwood M, McAlindon TE (2019). OARSI guidelines for the non-surgical management of knee, hip, and polyarticular osteoarthritis. Osteoarthr Cartil.

[4] Vanelli R, Costa P, Rossi SMP, Benazzo F (2010). Efficacy of intra-articular polynucleotides in the treatment of knee osteoarthritis: a randomised, double-blind clinical trial. Knee Surg Sports Traumatol Arthrosc.

[5] Saggini R, Di Stefano A, Cavezza T, Saladino G, Bellomo RG (2013). Intrarticular treatment of osteoarthropathy knee with polynucleotides: a pilot study with medium-term follow-up. J Biol Regul Homeost Agents.

[6] Giarratana LS, Marelli BM, Crapanzano C, De Martinis SE, Gala L, Ferraro M (2014). A randomized, double-blind clinical trial on the treatment of knee osteoarthritis: the efficacy of polynucleotides compared to standard hyaluronan viscosupplementation. Knee..

[7] Guizzardi S, Uggeri J, Belletti S, Cattarini G (2013). Hyaluronate increases polynucleotides effect on human cultured fibroblasts. J Cosmet Dermatol Sci Appl.

[8] Dallari D, Sabbioni G, Del Piccolo N, Carubbi C, Veronesi F, Torricelli P, Fini M (2020). Efficacy of intra-articular polynucleotides associated with hyaluronic acid versus hyaluronic acid alone in the treatment of knee osteoarthritis: a randomised, double-blind, controlled clinical trial. Clin J Sport Med.

[9] Kohn MD, Sassoon AA, Fernando ND (2016). Classifications in brief: Kellgren-Lawrence classification of osteoarthritis. Clin Orthop Relat Res.

[10] Insall JN, Dorr LD, Scott RD, Scott WN (1989). Rationale of the knee society clinical rating system. Clin Orthop Relat Res.

[11] Bellamy N, Buchanan WW, Goldsmith CH, Campbell J, Stitt LW (1988). Validation study of WOMAC: a health status instrument for measuring clinically important patient relevant outcomes to antirheumatic drug therapy in patients with osteoarthritis of the hip or knee. J Rheumatol.

[12] Gennero L, Denysenko T, Calisti GF, Vercelli A, Vercelli CM, Amedeo S, Mioletti S, Parino E, Montanaro M, Melcarne A, Juenemann C, Vivo ED, Longo A, Cavallo G, de Siena R (2013). Protective effects of polydeoxyribonucleotides on cartilage degradation in experimental cultures. Cell Biochem Funct.

[13] Fakhari A, Berkland C (2013). Applications and merging trends of hyaluronic acid tissue engineering, as a dermal filler and in osteoarthritis treatment. Acta Biomater.

[14] Bitto A, Polito F, Irrera N, D’Ascola A, Avenoso A, Nastasi G (2011). Polydeoxyribonucleotide reduces cytokine production and the severity of collagen-induced arthritis by stimulation of adenosine a(_2_A) receptor. Arthritis Rheum.

[15] Chung KI, Kim HK, Kim WS, Bae TH (2013). The effects of polydeoxyribonucleotides on the survival of random pattern skin flaps in rats. Arch Plast Surg.

[16] Daghestani HN, Kraus VB (2015). Inflammatory biomarkers in osteoarthritis. Osteoarthr Cartil.

[17] Heidari B, Hajian-Tilaki K, Babaei M (2016). Determinants of pain in patients with symptomatic knee osteoarthritis. Caspian J Intern Med.

[18] Akhtar N, Khan NM, Ashruf OS, Haqqi TM (2017). Inhibition of cartilage degradation and suppression of PGE2 and MMPs expression by pomegranate fruit extract in a model of posttraumatic osteoarthritis. Nutrition..

